# Characterization of spatial and temporal variations of CO_2_ concentration on tropical Island and analysis of influencing factors

**DOI:** 10.1038/s41598-025-32647-x

**Published:** 2025-12-13

**Authors:** Qi Luo, Jing Han, Shaojun Liu

**Affiliations:** 1 Hainan Institute of Meteorological Science, Haikou, 570203 China; 2 Sansha Marine Meteorology Field Experiment Station of CMA, Sansha, 573199 China; 3 South China Sea Marine Meteorology Hainan Observation and Research Station, Sansha, 573199 China

**Keywords:** Tropical island, CO_2_ concentration, Influencing factors, Variation trend, Climate sciences, Ecology

## Abstract

In this study, the spatial and temporal variations and distribution characteristics of the carbon dioxide (CO_2_) concentration on Hainan Island are analyzed using GOSAT L3 data from 2011 to 2024, and the effects of various factors impacting the CO_2_ concentration on Hainan Island are discussed. The results indicate that from 2011 to 2024, the CO_2_ concentration on Hainan Island showed an increasing trend, with a fast growth rate in the early period and a slow growth rate in recent years with the implementation of the dual-carbon strategy. The spatial distribution is affected by anthropogenic activities, topography, vegetation and solar radiation, and the overall CO_2_ concentration pattern is high in the north and low in the south. Human activities are the most important source of carbon on Hainan Island, vegetation is the most important carbon sink, and elements such as surface temperature, precipitation, and total solar radiation play roles in suppressing CO_2_. The CO_2_ concentration on Hainan Island is expected to continue to increase at a slow rate and may display a decreasing trend in the future.

## Introduction

Carbon dioxide (CO_2_) is among the most dominant greenhouse gases in the global atmosphere, and just over one hundred years after the Industrial Revolution, humans have burned the fossil energy accumulated by the Earth over billions of years. The burning of fossil fuels has resulted in the release of large amounts of CO_2_ into the atmosphere, while at the same time, the global vegetation area has been decreasing, and the concentration of CO_2_ in the atmosphere has been increasing rapidly^1^. The monthly average global CO_2_ concentration increased from 300 ppm before the start of the first industrial revolution to 410 ppm in April 2019, as measured by the National Oceanic and Atmospheric Administration (NOAA), representing a 40% increase in atmospheric CO_2_ concentration. The increase in atmospheric CO_2_ concentration is now the main cause of global warming, and the 2015 Paris Agreement, which set the goal of achieving net-zero emissions in the second half of the century, is being translated into national strategies by an increasing number of governments, with more than 130 countries and territories now proposing “zero-carbon” or “carbon-neutral” goals^2^. China’s carbon peak and carbon neutral strategy is not only a major demand for global climate governance, protection of Earth’s homeland and protection of the destiny of the human community but also an intrinsic demand for China’s high-quality development, ecological civilization and comprehensive ecological environment management^3^. The realization of the dual-carbon target has led to an urgent demand for the monitoring and assessment of the carbon cycle, especially carbon emission accounting. The regional carbon balance has also been a focus of global carbon cycle research in recent years^4–6^.

To control carbon emissions, it is first necessary to monitor their content; thus, monitoring the atmospheric CO_2_ concentration is important for studying the laws of the carbon cycle and addressing global warming^7^. At present, there are more than 300 greenhouse gas monitoring stations worldwide^8^. Ground-based observations have advantages such as high precision, high reliability, and real-time access, but the results represent only single-point measurements, and macro- and vertical detection capabilities are lacking. Satellite remote sensing detection is important for the study of large-scale variations in CO_2_ concentrations^9,10^. Satellite remote sensing technology involves detecting and receiving information from target objects through sensors at high altitudes^11^ to identify the attributes, spatial distribution and other characteristics of objects; satellite data are acquired and analyzed through remote sensing technology platforms. The detection capability of satellite remote sensing depends mainly on the on-board detection instruments and the remote sensing inversion algorithms. The shortwave infrared band, which is sensitive to variations at the ground level, is suitable for monitoring the dynamic changes in carbon sources and sinks on the ground. The SCIAMACHY instrument on board the ENVISAT satellite was the first on-board detector to use the shortwave infrared absorption band as its detection band^12^. Subsequently, multiple carbon monitoring satellites, such as the Greenhouse Gas Observing Satellite (GOSAT) launched by Japan^13^, the Orbiting Carbon Observing Satellite-2 (OCO-2) launched by the United States, and China’s Chinese Carbon Satellite (TanSat), which was launched in 2017, have utilized this detection band, and the detection accuracy has gradually increased with the continuous improvement of detector indicators and inversion methods. Satellite remote sensing detection has advantages such as large ranges, long time series, and vertical detection and these advantages allow for the can improved assessment of the spatial and temporal distribution and change characteristics of global CO_2_ concentrations^14^; many scholars have also studied CO_2_ concentrations using satellite data: Yokota T, et al. utilized preliminary GOSAT data to analyze the distribution of global CO_2_ and CH_4_^15,16^; Heyntann J, et al. presented the first detailed assessment of the new GOSAT BESD X CO_2_ product^12^; a model based on temperature (MOD11C3), vegetation cover (MOD13C2 and MOD15A2) and productivity (MOD17A2) MODIS products was developed by Guo M et al.^17^ to assess CO_2_ concentrations at a global scale. It is verified that the GOSAT data can better capture the characteristics of the variation of CO_2_ concentration in the near-surface observation data, and the comparison study with the observation data from the atmospheric background station at Wariguan shows that the accuracy and stability of the GOSAT data are high.

Hainan Island (18°09′~20°11′N, 108°37′~111°03′E) is located in the southernmost part of China and has a typical tropical monsoon climate^18^. The terrain is high in the center and low in the surrounding area, with the highest mountain range located in the area surrounding Wuzhi Mountain; Wuzhi Mountain and Parrot Ridge represent the core of the uplift, with the elevation descending in a stepwise manner toward the periphery, and the terrain consists of a circular layered landscape of mountains, hills, plateaus, and plains, with an obvious gradient structure. Hainan Island is an important distribution area for tropical and monsoon rainforests and has a variety of natural vegetation, such as evergreen broad-leaved forests, mangrove forests and coniferous forests^19^. At present, few studies have investigated the distribution and influencing factors of CO_2_ concentration on Hainan Island^20^, and there are few CO_2_ monitoring stations on Hainan Island^21^. Satellite data have become important for studying CO_2_ concentrations on Hainan Island. In view of this, in this study, the multi-year changes in and distribution of the CO_2_ concentration on Hainan Island were first analyzed by using GOSAT satellite data; second, a variety of natural and anthropogenic driving factors were selected, and the influences of the different driving factors on the CO_2_ concentration on Hainan Island were investigated; then, the evolutionary characteristics of the CO_2_ concentration on Hainan Island were discussed. The evolution of the CO_2_ concentration on Hainan Island is evaluated to better understand the patterns of the atmospheric CO_2_ concentration under the unique climate background of Hainan Island, which will promote an improved understanding of the mechanisms to control atmospheric CO_2_ concentrations.

## Results

### Characteristics of CO_2_ concentrations on Hainan Island from 2011 to 2024

**Fig. 1:**
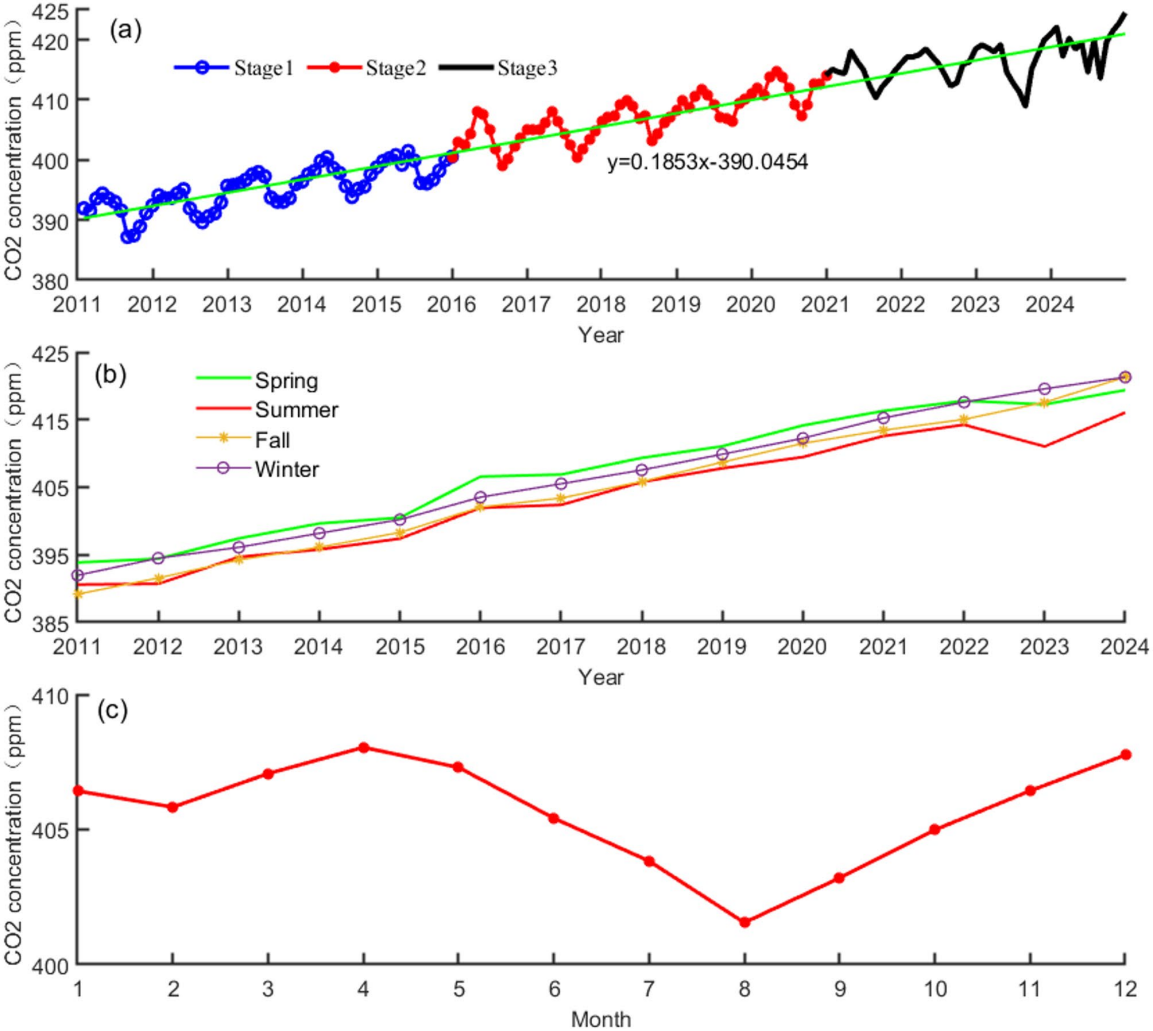
Annual variations in CO_2_ concentration (**a**), seasonal variations in CO_2_ concentration (**b**) and monthly mean CO_2_ concentrations (**c**) on Hainan Island from 2011–2024.

As shown in Fig. 1a, the CO_2_ concentration on Hainan Island from 2011 to 2024 tends to increase annually, from 391.39 ppm at the beginning of 2011 to 419.51 ppm at the end of 2024, with an average growth rate of 2.01 ppm/y, which is lower than the average worldwide growth rate over the past 10a of 2.06 ppm/y proposed by the World Meteorological Organization. The growth rate of the CO_2_ concentration can be divided into three phases. The first stage is 2011–2015, during which the CO_2_ concentration growth rate is relatively slow, except for 2012–2013, when the growth rate of the CO_2_ concentration is less than 2.0 ppm/y, with an average growth rate of 1.92 ppm/y; the second stage is 2015–2021, when the CO_2_ concentration on Hainan Island increases rapidly, with an average growth rate of 2.55 ppm/y, of which the greatest growth rate reached 4.45 ppm/y in 2015–2016, probably because of the impact of the strong El Niño phenomenon in 2015^22^. In the third phase, from 2021 to 2023, with the implementation of the “carbon peak and carbon neutral” strategy, the growth rate of the CO_2_ concentration slowed significantly in the last two years, with rates of 1.78 and 0.20 ppm/y, respectively.

The seasonal variations in the CO_2_ concentration are shown in Fig. 1b. In the winter, vegetation cover is low, and the vegetation leaf area is small; thus, photosynthesis is limited, and the CO_2_ concentration is high. In spring, the weather becomes warmer, and vegetation and soil respiration increase; moreover, as the temperature increases, soil microbial activity is strengthened to decompose CO_2_ in soil biomass^23^, so the CO_2_ concentration reaches the highest level, with an average value of 407.47 ppm; in contrast, the vegetation coverage is highest in summer, when the leaf area increases, photosynthesis is the strongest, and more CO_2_ can be absorbed from the atmosphere, so the CO_2_ concentration is the lowest in summer, with an average value of 402.47 ppm. In the fall, photosynthesis is weakened, and the CO_2_ concentration gradually increases. Owing to the unique tropic climatic conditions, the temperature difference between summer and winter is relatively small. The difference in CO_2_ concentrations between summer and fall on Hainan Island is relatively small too. As shown in Fig. 1c, the average monthly CO_2_ concentration on Hainan Island increased from January to April, with April having the highest value of the year, reaching 408.04 ppm, and then gradually decreasing from April to August with increasing photosynthesis, and the CO_2_ concentration in August was 400.63 ppm, which was the lowest value of the year. From August to December, the vegetation leaf area gradually decreased; thus, the chlorophyll content decreased. The uptake of CO_2_ by photosynthesis decreased and the CO_2_ concentration gradually increased again. The greatest difference between seasons occurred from spring to summer, with an absolute difference of 4.81 ppm, followed by 2.62 ppm from fall to winter; the differences from winter to spring and from summer to spring were similar to each other, with the absolute value of the former (1.22 ppm) being slightly greater than that of the latter (1.00 ppm); the values in spring and summer were much greater than those in other seasons, which indicates that terrestrial vegetation ecosystems play a significant role as carbon sinks.

### Characteristics of the Spatial distribution of CO_2_ concentration on Hainan Island

**Fig. 2:**
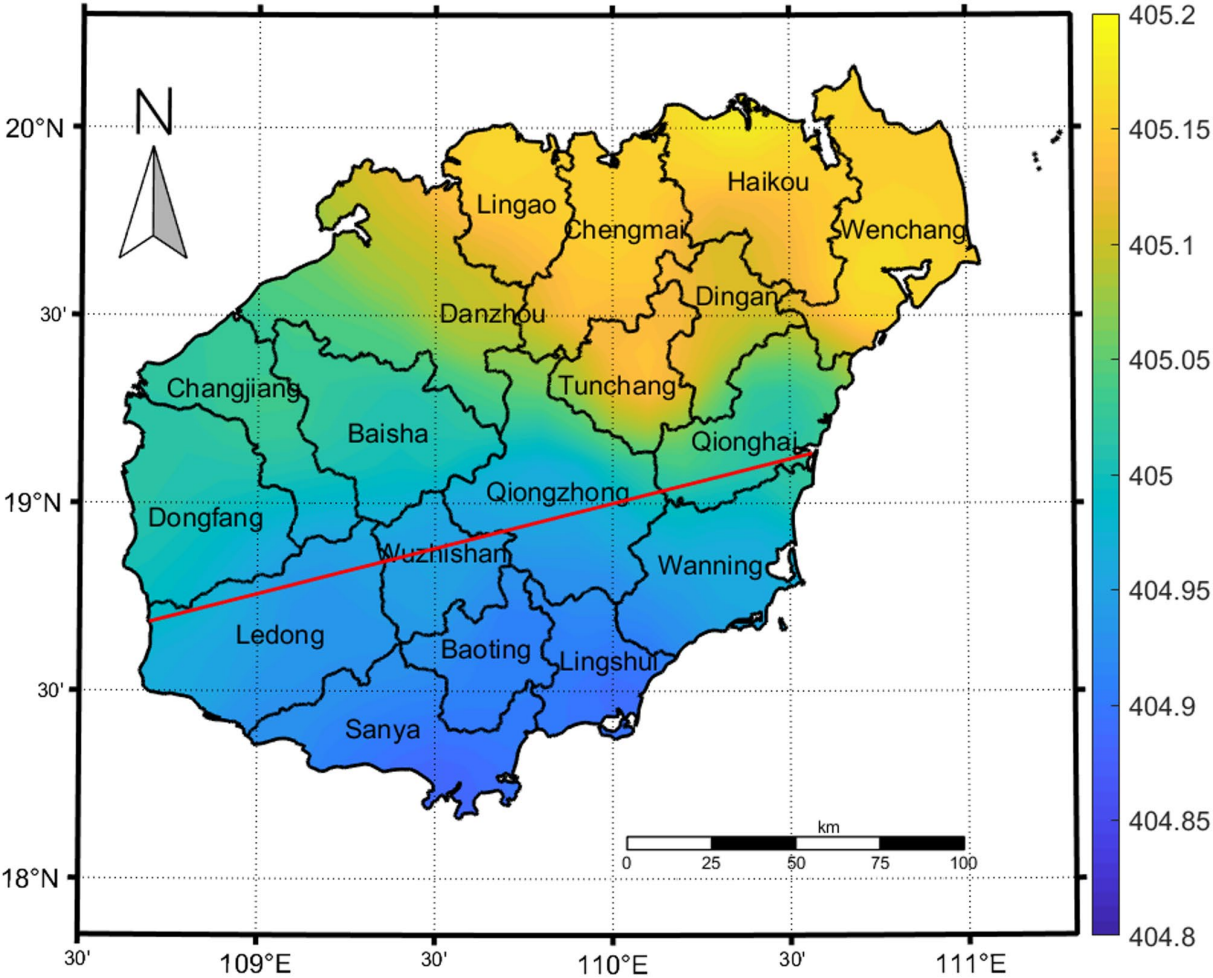
Multiyear average distribution of CO_2_ concentrations on Hainan Island, 2011–2024. This map was generated by the authors using MATLAB software (Version R2024a, https://matlab.mathworks.com/).

**Fig. 3:**
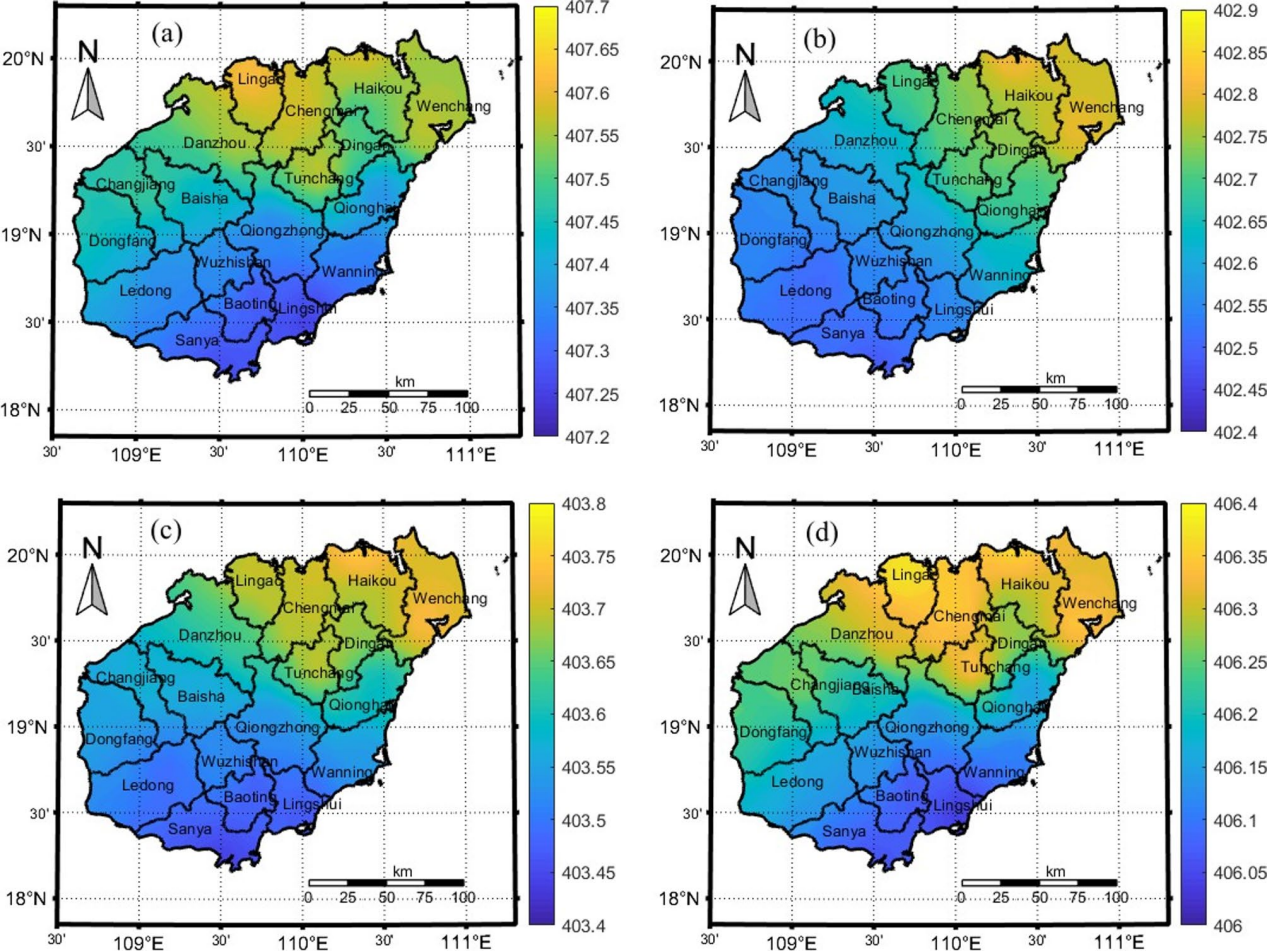
Distribution of CO_2_ concentrations on Hainan Island during different seasons. This map was generated by the authors using MATLAB software (Version R2024a, https://matlab.mathworks.com/). (**a**) Spring (**b**) Summer (**c**) Fall (**d**) Winter.

The distribution of the average CO_2_ concentration on Hainan Island is shown in Fig. 2, which shows clear differences in the CO_2_ concentrations in the north and south with high concentrations in the north and low concentrations in the south; the highest value occurs in the capital city of Haikou, reaching 405.19 ppm, and the lowest value occurs in Sanya, reaching 404.88 ppm. There is a clear demarcation line in the central part of the island, where the concentration gradually increases in the north starting in Wuzhishan and decreases in the south. In the north, Haikou, Lingao, Wenchang and Chengmai have high CO_2_ concentrations that tend to decrease to the south, and the concentrations decrease toward the south. The main reason for this phenomenon is that the highest peaks on Hainan Island are mostly located in the Wuzhishan and Parrot Mountain ranges in the central part of the island, whereas the terrain in the south-central part of the island is characterized by mountainous and hilly areas, which block the advection and turbulent diffusion of CO_2_ to the south. The seasonal CO_2_ concentration in each city and county was in the order of spring > winter > fall > summer, and there were no obvious differences in the spatial distribution of the CO_2_ concentration in each season; the overall pattern was high in the north and low in the south (Fig. 3). The high-value area in the north covers the widest range in winter, followed by spring and fall, and the high-value area covers the smallest range in summer and is concentrated around Haikou and Wenchang in the northeast.

### Analysis of factors affecting the CO_2_ concentration on Hainan Island

CO_2_ concentration is affected by various factors, such as vegetation photosynthesis, population growth, and industrial emissions. In this study, natural factors, such as the enhanced vegetation index, surface temperature, precipitation, and solar radiation, and anthropogenic factors, such as the population, GDP, and energy consumption of each city and county of Hainan Island, were selected and analyzed at both monthly and yearly scales (of which only yearly averages were available for population, GDP, and energy consumption).

### Effects of anthropogenic activities on the CO_2_ concentration on Hainan Island

**Fig. 4:**
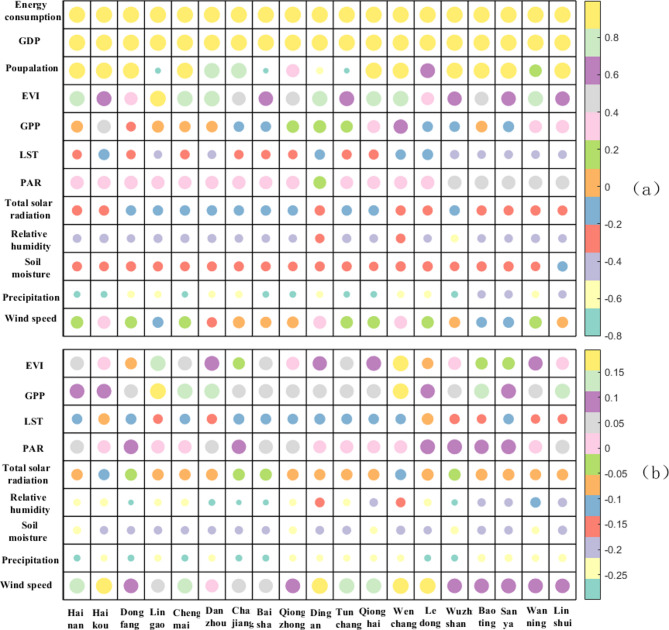
Correlations between CO_2_ concentrations and driving factors in cities and counties on Hainan Island. (**a**) Annual scale (**b**) Monthly scale.

On the annual scale, the GDP and energy consumption (coal, oil, natural gas, and primary electricity) of the cities and counties on Hainan Island were significantly positively correlated with the CO_2_ concentration, with correlation coefficients of 0.9 or greater(Fig. 4). Population was also significantly positively correlated with CO_2_ concentration in all counties except for Ding’an, Lingao, Tunchang, and Baisha, mainly because the population of these counties has been declining in recent years. Overall, the population, GDP and energy consumption continue to increase and play important roles in promoting the CO_2_ concentration on Hainan Island, as these are the main sources of carbon on the island. Before 2020, the population, GDP, and energy consumption all grew rapidly, resulting in a fast rate of increase in the CO_2_ concentration. After 2020, with the slowdown of population growth, the GDP and energy consumption both also decreased, and the CO_2_ concentration decreased.

### Influence of natural factors on the CO_2_ concentration on Hainan Island

The correlation between each natural factor and the CO_2_ concentration at the annual scale is high(Fig. 4a), and the variations at the monthly scale are relatively smooth(Fig. 4b), compared with those at the annual scale. The greatest inhibition of CO_2_ concentration on Hainan Island is associated with moisture, precipitation, soil moisture and relative humidity, which are negatively correlated on both the annual and monthly scales, and the correlation between CO_2_ concentration and precipitation is greatest in each city and county at the annual scale. Surface temperature affects vegetation growth and human activities, and surface temperature is affected mainly by the intensity of solar radiation, which in turn affects ecosystem respiration and photosynthesis. Surface temperature and total solar radiation also significantly inhibited the CO_2_ concentration. The correlation of surface temperature with CO_2_ concentration was second only to that of precipitation on the annual scale; however, the correlation lower on the monthly scale than on the annual scale, which was attributed to Hainan Island being located in the tropics and the surface temperature varying little throughout the year. Wind drives turbulence in the atmosphere, and a pressure gradient activates the CO_2_ dispersal.High wind speeds can accelerate the dilution and transportation of CO2 in the air and reduce the local concentration; the higher the wind speed is, the more uniform the distribution of CO_2_ is in the atmosphere, which reduces the occurrence of localized areas with high concentrations; strong winds can also drive the vertical transport of CO_2_ and change its concentration in different atmospheric layers, but the mean wind speed at both the monthly and yearly scales was low and thus did not greatly impact the CO_2_ concentration.

The enhanced vegetation index (EVI) is an important indicator used in remote sensing to monitor vegetation growth and cover, and its sensitivity is better than that of other vegetation indices for areas with high vegetation cover^24^. Photosynthetically active radiation (PAR) refers to the band of solar radiation that can be used by plants for photosynthesis and is a key energy source during the light reaction stage. PAR directly affects the photosynthetic rate, plant growth and ecosystem productivity. The correlations between the CO_2_ concentration and the EVI and PAR in the cities and counties of Hainan Island were not significant and were weakly positive. On the one hand, Hainan Island is in a tropical area, and the average annual temperature is high. The pillar industries are tourism and tropical agriculture, with limited heavy industry, and the annual vegetation cover is high and has been on the rise in recent years. The average annual EVI value is greater than the average level in China; compared with the sparsely vegetated areas where vegetation significantly increases the role of CO_2_, the effect of vegetation on the concentration of CO_2_ on Hainan Island is limited. On the other hand, with increasing global average temperature, the amount of CO_2_ produced by vegetation respiration may be underestimated, especially under high-temperature and high-humidity conditions on Hainan Island, and the activity of organic matter in the soil is enhanced, resulting in increased CO_2_ emissions. In addition, long-term high temperatures in the tropics are accompanied by drought stress, and plants close their stomatal pores to reduce water transpiration under these conditions^25,26^. Moreover, high temperature accelerates the degradation of chlorophyll and reduces the ability of plants to capture light, resulting in limited CO_2_. uptake by vegetation.

**Fig. 5:**
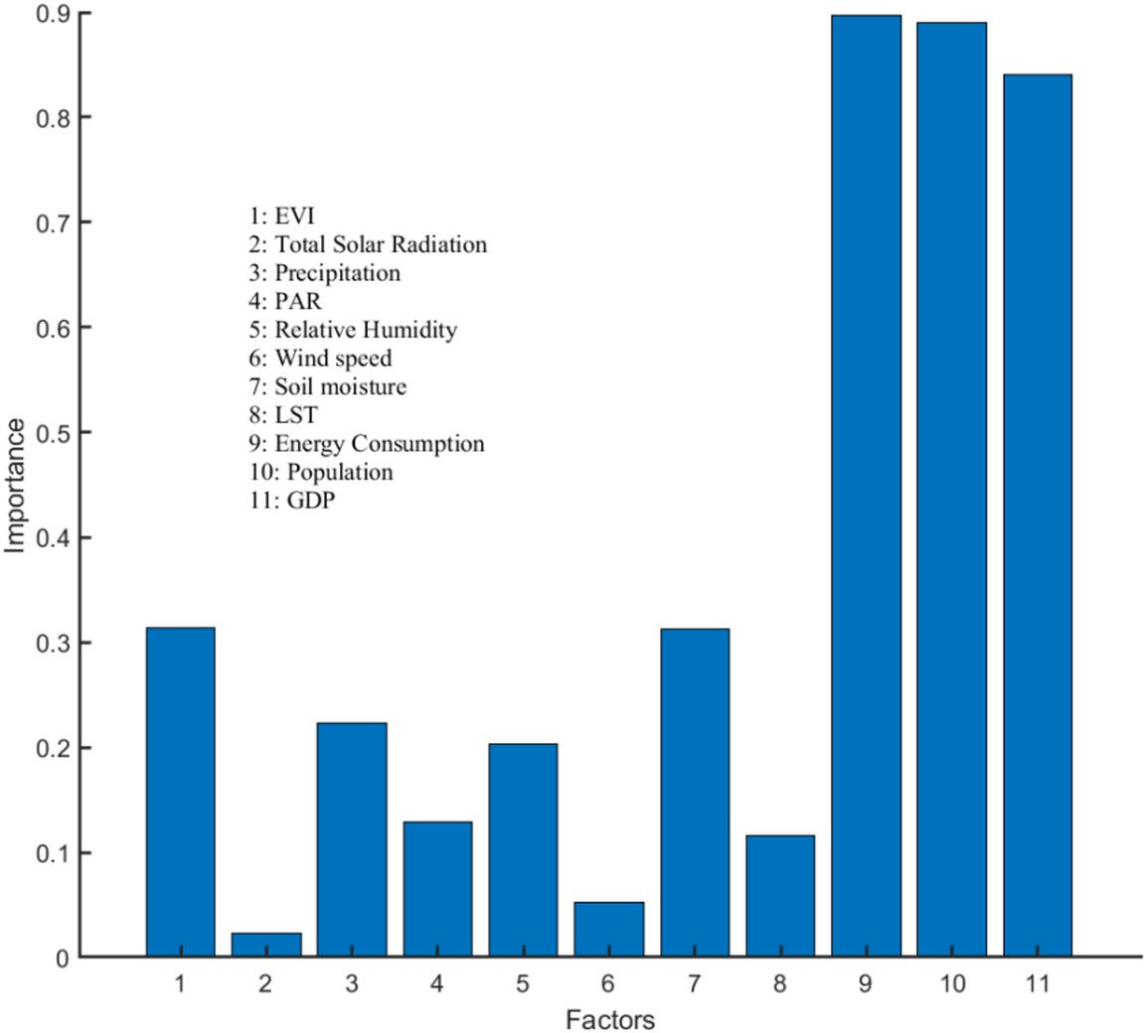
Factor importance based on random forest.

Figure 5 shows the random forest importance of driving factors. The results indicate that socio-economic factors are the most influential driving factors.The importance values of population、energy consumption and GDP are significantly higher than other factors, which are close to 0.9. Medium importance factors with values ranging from approximately 0.1 to 0.4 includes EVI、LST 、Soil moisture、Precipitation、Relative humidity and PAR. The total solar radiation and wind speed have the least influence on CO_2_ concentration.

#### Combined impact analysis of multiple drivers

The CO_2_ concentration is jointly influenced by multiple driving factors. Based on the multiple regression analysis method, comprehensive impact analysis was conducted on several driving factors that have significant impact on CO_2_ concentration . Due to the large difference in magnitude between the factors, the data were normalized, and we obtained the contributions of the different driving factors to the impact on the CO_2_ concentration on Hainan Island in each season. Table 1 shows that the seasonal changes of CO_2_ concentration on Hainan Island is significantly affected by different driving factors. Although vegetation is not sensitive to changes in CO_2_ concentration on Hainan Island, it is still the most important carbon sink, especially in the summer when vegetation photosynthesis is the strongest. In addtion, the vegetation undergoes decomposition during the autumn/winter season, and some of the carbon goes back into the atmosphere through biomass decomposition. The control effect of PAR on the CO_2_ concentration reaches the maximum value with a regression parameter of −0.218 in summer, which is much greater than that in the other seasons. Relative humidity and surface temperature both inhibited the CO_2_ concentrations, whereas precipitation had an inhibitory effect on the CO_2_ concentration but had a small contribution to the regression equation. Energy consumption (E) is the most important carbon source, and the regression parameter is high in all seasons, with the highest being 0.936 in spring. The coefficient of determination of the relative regression equation(R^2^) is greater than 0.75 in all seasons, which represents a good fit. The Variance Inflation Factor (VIF) of each variable is less than 5, indicating that there is no multicollinearity issue among the independent variables (Table 2).

**Table 1:** Seasonal contributions of different driving factors to the variation in CO_2_ concentration on Hainan Island.

	EVI	PAR	LST	PRE	RH	E	R2
spring	0.081	0.104	−0.006	0.149	−0.228	0.936	0.776
summer	−0.104	−0.218	−0.051	0.077	−0.261	0.741	0.753
fall	−0.118	−0.116	−0.086	0.003	−0.16	0.81	0.884
winner	0.217	0.114	−0.072	0.022	0.056	0.906	0.894

**Table 2:** Variance inflation factor (VIF) of driving factors.

	EVI	PAR	LST	PRE	RH	E
spring	3.989	3.780	2.646	3.710	3.545	1.601
summer	2.213	3.512	3.191	3.260	3.846	1.658
fall	4.186	4.542	5.840	3.875	1.950	1.777
winner	1.822	2.791	2.945	1.293	2.693	1.346

### Trend analysis of the CO_2_ concentration on Hainan Island

**Fig. 6:**
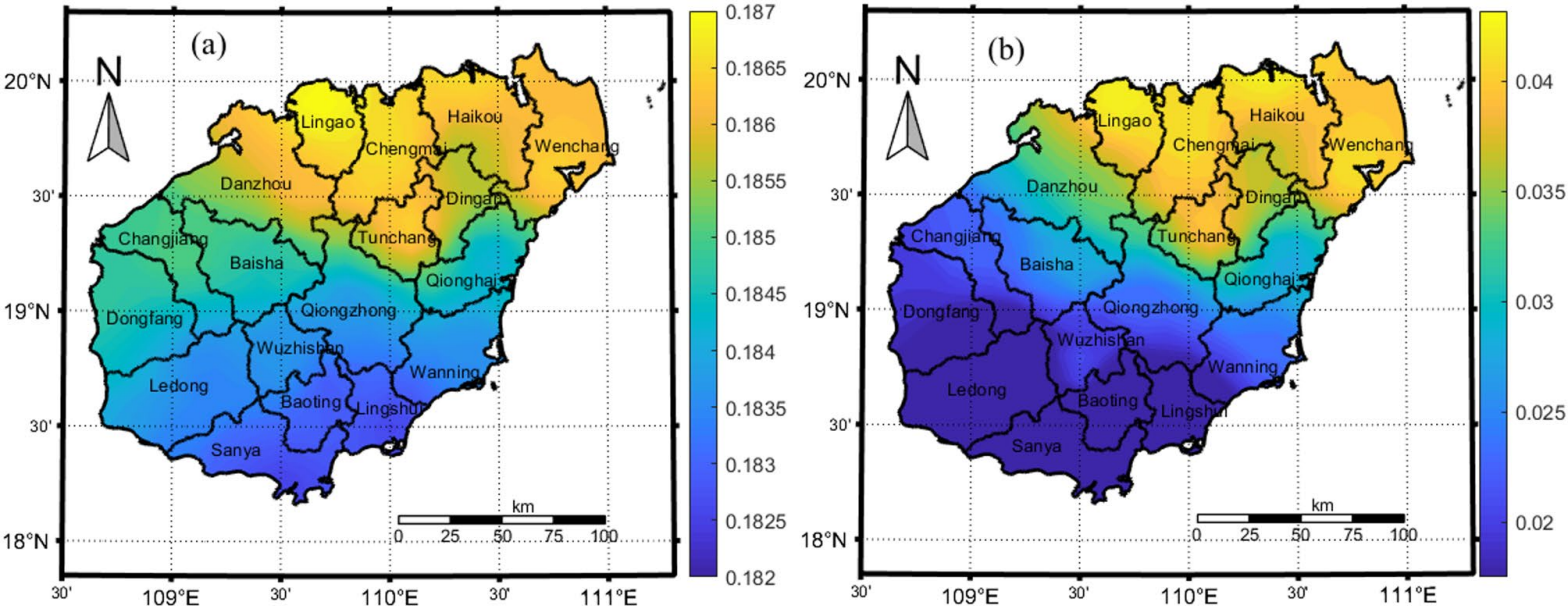
Spatial evolution of the CO_2_ concentration on Hainan Island. This map was generated by the authors using MATLAB software (Version R2024a, https://matlab.mathworks.com/). (**a**) Trend analysis (**b**) R/S analysis (Hurst index).

The trend of the CO_2_ concentration on Hainan Island is shown in Fig. 6a, which shows the slope of the regression equation for the CO_2_ concentration, with a positive value indicating an increasing trend, larger values indicate greater rates of increase. The slope values for the cities and counties on Hainan Island are small and do not substantially differ, especially between Haikou (0.187) and Sanya (0.182), which means that the CO_2_ concentration in the cities and counties will continue to increase at a relatively slow rate.

The results of the stability analysis of the spatial evolution of CO_2_ concentration growth on Hainan Island based on the R/S analysis are shown in Fig. 6b. An H value (Hurst index) between 0 and 0.5 indicates that the CO_2_ concentration time series exhibits inverse persistence, the past variables are negatively correlated with the future trend. The CO_2_ concentration time series with values of 0.5 < H < 1 exhibits a long-term correlation, and the process is continuous. As shown in the Fig. 6b, the H values are less than 0.5 in all cities and counties on Hainan Island, which indicates that the CO_2_ concentration time series is strongly inversely persistent and that the CO_2_ concentration may decrease in the future.

### Future CO_2_ concentration forecast for Hainan Island

**Fig. 7:**
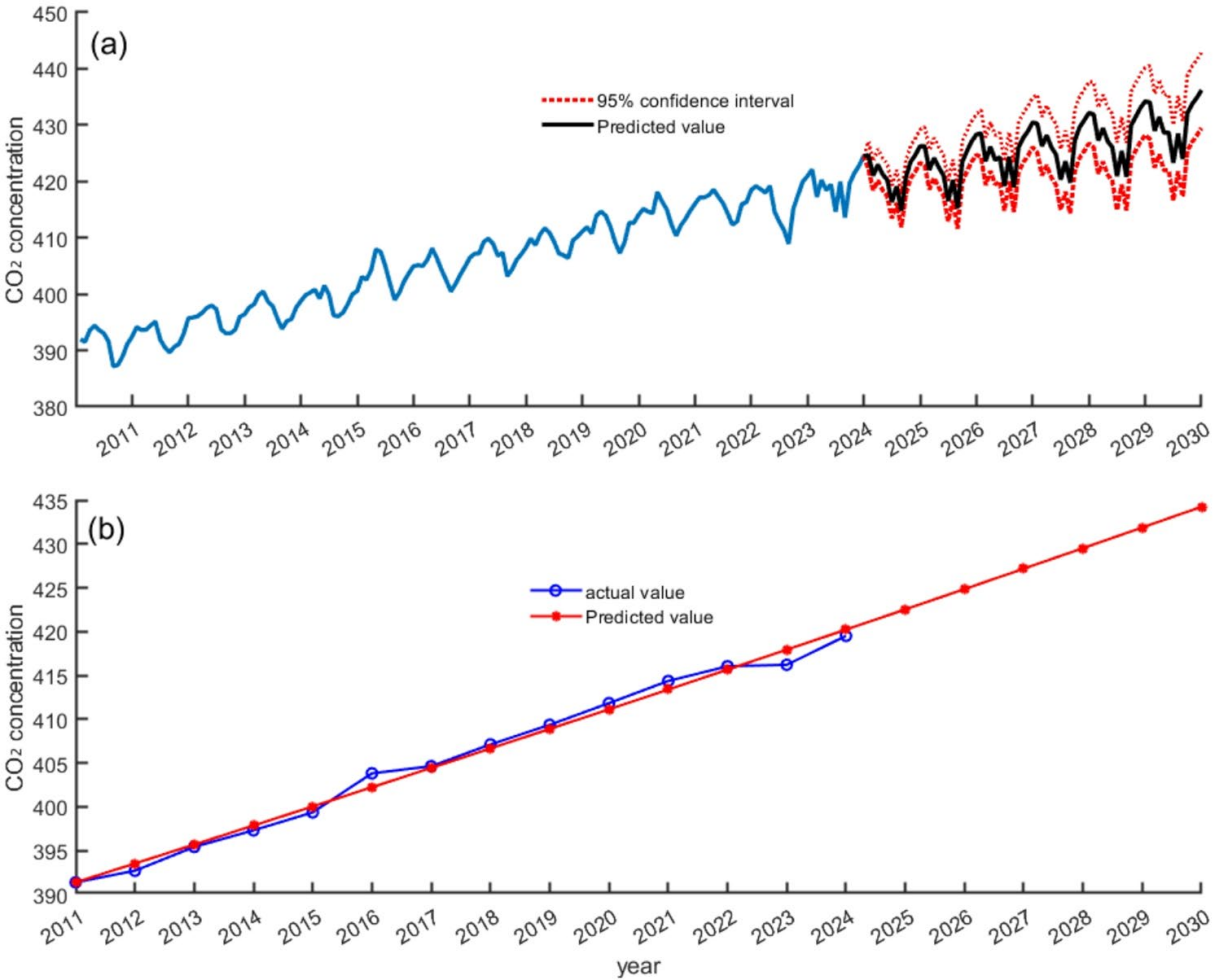
Trends in CO_2_ concentrations on Hainan Island for 2025 to 2030. (**a**) SARIMA model (**b**) Gray model.

The gray model is suitable for scenarios with little data and obvious trends^27^, whereas the SARIMA model is suitable for scenarios with obvious seasonality^28^. Therefore, the interannual and monthly changes in CO_2_ concentration on Hainan Island for 2025 to 2030 were predicted using the gray model and the SARIMA model, respectively. The results show that the interannual (Fig. 7a) and monthly (Fig. 7b) changes in CO_2_ concentrations on Hainan Island in the future will show an increasing trend, but the growth rate may be lower than the global average, which is consistent with the conclusion in Sect. 2.3.4.

## Discussion

1. 3.1 Limitations of the study: The GOSAT L3 data used in this study have a relatively coarse native spatial resolution. Although the data were downscaled using kriging interpolation, the ability to use this product to resolve spatial differences within Hainan Island is limited. The interpolation results primarily reflect the estimated distribution of localized driving factors (such as population distribution and topography) over a large-scale background. The uncertainty of these results is difficult to quantify without verifications from ground-based observations.
2. 3.2 The distribution of the average CO_2_ concentration on Hainan Island is consistent with the distribution of the population and economy. The northern part of the island (Haikou and Wenchang) is the most populated area, with intensive industrial, tourism and international shipping activities and a high socioeconomic level, with the population and GDP accounting for 55% and 60% of the island total, respectively; therefore, the CO_2_ concentrations due to anthropogenic activities are relatively high. The south-central part of Hainan Island has the largest tropical rainforest, accounting for 1/7 of the island area, with a strong carbon sink capacity and relatively sparse population; thus, the impact of anthropogenic activities is small in this region. The central part of the island lags behind the southern coastal area in terms of population and economy, but the average CO_2_ concentration is higher than that in the southern coastal area, which also indicates that the spatial distribution of CO_2_ is affected not only by anthropogenic activities but also by the long-distance transportation of CO_2_ caused by atmospheric circulation patterns that are influenced by topography, vegetation and nonuniform distribution of solar radiation on the Earth’s surface.
3. 3.3 Background of a global increase in CO_2_ concentrations of 2–3 ppm/y. Despite the relatively slow growth rate in CO_2_ concentrations on Hainan Island, it remains difficult for the region to escape the global trend of increasing CO_2_ concentrations as influenced by the global atmospheric cycle. To achieve the “zero carbon” and “carbon neutral” goals at an early date, the following recommendations are proposed:

(1) Promotion of the green transformation of industries. Energy-saving transformation in the petrochemical and cement industries should be promoted. Low-carbon transformation of tropical agriculture and tourism should be carried out to reduce total carbon emissions.

(2) Increasing the utilization rate of renewable energy. The use of new energy vehicles(electric) should be promoted. The geographical advantages of the South China Sea should be leveraged to accelerate the large-scale development of offshore wind power and distributed photovoltaics.

(3) The ecological protection of the central tropical rainforest should be strengthened, the scale of the restoration of mangrove forests, seagrass beds and coral reefs should be expanded, and ecological carbon sinks should be enhanced.

(4) Greenhouse gas observation systems should be deployed in key areas of Hainan Island and the South China Sea and the carbon sink capacities of land and ocean areas should be assessed and monitored.

## Conclusions

(1) The CO_2_ concentrations on Hainan Island from 2011 to 2024 tended to increase annually, with an average growth rate of 2.01 ppm/y; the growth rate increased from 2015 to 2021 when the average growth rate was 2.55 ppm/y; the greatest rate increase occurred from 2015 to 2016, reaching 4.45 ppm/y, and the smallest rate increase of 0.20% occurred in 2022–2023. The CO_2_ concentration on Hainan Island shows obvious seasonal variations, following the pattern of spring > winter > fall > summer, with April having the highest value in the year, reaching 408.04 ppm. The CO_2_ concentration in August was the lowest throughout the year, at 401.55 ppm. The greatest difference between seasons occurred from spring to summer, with an absolute value of 4.81 ppm.

(2) The spatial distribution of CO_2_ on Hainan Island is affected by anthropogenic activities, topography, vegetation and solar radiation, and the overall pattern is high in the north and low in the south; moreover, there is no obvious difference in the spatial distribution of CO_2_ across seasons. Human activities are the most important carbon source on Hainan Island. Vegetation is insensitive to changes in CO_2_ concentration on Hainan Island but remains the most important carbon sink, and elements such as surface temperature, precipitation, and total solar radiation also play a role in suppressing the CO_2_ concentration.

(3) The trend of the CO_2_ concentration shows that the slope values of each city and county on Hainan Island are small and do not differ much, and the CO_2_ concentration will continue to increase at a relatively slow rate. The H value (Hurst index) of each city and county is less than 0.5, which indicates that the CO_2_ concentration time series exhibits strong inverse persistence and that the CO_2_ concentration may show a decreasing trend in the future. The gray model and SARIMA model were used to predict the CO_2_ concentration on Hainan Island from 2025 to 2030, and the results also revealed that the CO_2_ concentration on Hainan Island will continue to increase at a slow rate.

## Methods

### Research data

GOSAT is a greenhouse gas observation satellite launched by Japan in 2009, and it is also the world’s first satellite dedicated to the observation of greenhouse gases. the observation performance of GOSAT has made a great leap compared with the early SCIAMACHY, the revisit period of GOSAT is only 3 days, the spatial resolution has been greatly improved from the original 30 * 60 to 10.5 * 10.5. GOSAT is a satellite with a short-wave near-infrared (NIR) band, which is sensitive to the change of CO2 content in the near-surface layer, and it can effectively obtain the whole layer of CO2 information, including the bottom atmosphere14. This study uses the L3-level data of GOSAT satellite, which mainly includes the monthly average CO2 concentration with a spatial resolution of 2.5°*2.5°. The Enhanced Vegetation Index (EVI), Gross Primary Productivity (GPP), Photosynthetically Active Radiation (PAR) and LST data were obtained from MODIS (Moderate-Resolution Imaging Spectroradiometer), a large-scale space-based remote sensing instrument developed by NASA24. The MODIS (Moderate Resolution Imaging Spectroradiometer) is a large spaceborne remote sensing instrument developed by NASA (National Aeronautics and Space Administration), which is characterized by free access, a wide spectral range (36 bands, from visible to thermal infrared), and a high updating frequency (at least twice a day with global coverage), and the raw raster data were averaged according to the administrative boundaries of the prefectures and municipalities. Total solar radiation, Relative humidity, precipitation and wind speed data were obtained from the ERA5-Land reanalysis dataset from the European Center. ERA5-Land uses the laws of physics to combine modeled data with observations from around the world to form a globally complete and consistent dataset^29^, with a spatial and temporal resolution of 0.1°*0.1° per month. Population, GDP, and energy data were obtained from the Statistical Yearbook of Hainan Province.

### Kriging interpolation

Kriging interpolation (Kriging) is a statistically based spatial interpolation method, which is capable of generating a more accurate spatial prediction model by considering spatial autocorrelation and the weights of the data points.Kriging interpolation not only takes into account the positional relationship between the observation points and the estimation points, but also integrally takes into account the relative positional relationship between the observation points, and it has been widely used in spatial statistical analysis^30^. In this study, the monthly data of spatially resolved CO2 concentration at 0.1°*0.1° on Hainan Island from 2011 to 2024 are obtained by kriging interpolation using GOSAT L3 2.5°*2.5° data.

### R/S analysis

R/S analysis, also known as Rescaled Range Analysis, is a statistical analysis method first proposed by hydrologist H.E. Hurst in 1951, which is mainly used to study the long-range correlation of time series data.R/S analysis evaluates the statistical characteristics of a time series by calculating the R/S ratio of the time series, i.e., the ratio of the range to the standard deviation^31^. R/S analysis assesses the statistical properties of time series, especially by analyzing the H-value (Hurst’s index) to determine the trend persistence or anticontinuity of the time series, and the method is also applied to geography, climatology, etc. to study the long-term changes and forecasts of natural phenomena.

### Gray and SARIMA models

Grey forecasting and Grey Model (GM) are the core forecasting methods in Grey Systems Theory, which are applicable to the forecasting of small samples and uncertain systems. The most commonly used is the GM(1,1) model (first-order univariate gray model), which is suitable for short-term forecasting of time series data.The SARIMA model (Seasonal Autoregressive Integral Sliding Average Model) is an extension of the ARIMA model, which is specifically used to deal with the time series data (such as monthly and quarterly data) with a seasonal cycle, and the core idea of which is to add seasonal difference and seasonal autoregressive/moving average terms to ARIMA.

## References

[1] The state of. Greenhouse gases in the atmosphere based on global observations through 2010. *WMO Greenh. Gas Bull.* **7**(21) (2011).

[2] Zhang, S. et al. Policy recommendations for the zero energy building promotion towards carbon neutral in Asia-Pacific Region. *Energy Policy*10.1016/j.enpol.2021.112661 (2021).

[3] Gregg, J. S., Andres, R. J., Marland, G. & China Emissions pattern of the world leader in CO_2_ emissions from fossil fuel consumption and cement production. *Geophys. Res. Lett.* **35** (8), 135–157 (2008).

[4] Etheridge, D. M. et al. Natural and anthropogenic changes in atmospheric CO_2_ over the last 1000 years froin air in Antarctic ice and firn. *J. Geophys. Research: Atmos.* **101**(D2), 4115 (1996).

[5] Li, C., Li, H. & Qin, X. Spatial heterogeneity of carbon emissions and its influencing factors in china: evidence from 286 prefecture-level cities. *Int. J. Environ. Res. Public Health*. **19** (3), 1226 (2022). 35162249 10.3390/ijerph19031226 PMC8834810

[6] Zhang, M. N. et al. Elevated CO_2_ moderates the impact of climate change on future bamboo distribution in Madagascar. *Sci. Total Environ.* **810**, 152235 (2022). 34890677 10.1016/j.scitotenv.2021.152235

[7] Jung, M. et al. Compensatory water effects link yearly global land CO_2_ sink changes to temperature. *Nature* **541** (7638), 516–520 (2017). 28092919 10.1038/nature20780

[8] Yamagishi, H. et al. Role of nitrification and denitrification on the nitrous oxide cycle in the Eastern tropical North Pacific and Gulf of California. *J. Geophys. Research:Biogeosciences*. 10.1029/2006JG000227 (2007).

[9] Humphrey, V. et al. Sensitivity of atmospheric CO_2_ growth rate to observed changes in terrestrial water storage. *Nature* **560** (7720), 628–631 (2018). 30158603 10.1038/s41586-018-0424-4

[10] Yi, L. I. U. et al. Advances in technologies and methods for satellite remote sensing of atmospheric CO2. *Remote Sens. Technol. Application*. **26** (2), 247–254 (2011).

[11] Wang, Q., Chiu, Y. H. & Chiu, C. R. Driving factors behind carbon dioxide emissions in china: A modified production-theoretical decomposition analysis. *Energy Econ.* **51**, 252–260 (2015).

[12] Heyntann, J. et al. Consistent satellite X CO_2_ retrievals from SCIAMACHY and GOSAT using the BESD algorithm. *Atmos. Meas. Tech.* **8** (2), 2961–2980 (2015).

[13] Takagi, H. et al. On the benefit of GOSAT observationsto the Estimation of regional CO_2_ fluxes. *Sola* **7**, 161–164 (2011).

[14] Buchwitz, M. et al. Atmospheric Methaneand carbon dioxide from SCIAMACHY satellite data: initial comparison with chemistry and transportmodels. *Atmos. Chem. Phys.* **5** (4), 941:962 (2005).

[15] Yizhen, J. I. A. et al. Spatial and Temporal distribution of XCO_2_ and XCH_4_ in China based on satellite remote sensing. *J. Atmospheric Environ. Opt.* **17** (6), 679692 (2022).

[16] Yokota, T. et al. Global concentrations of CO_2_ and CH4 retireved from GOSAT: first preliminary results. *Sola* **5**, 160163 (2009).

[17] Guo, M. et al. Assessment of Global Carbon Dioxide Concentration Using MODIS and GOSAT Data. *Sensors*10.3390/s121216368 (2012). 23443383 10.3390/s121216368 PMC3571787

[18] Da-zhang, H. E. & Sheng-ling, Z. H. A. N. G. The Reginal climate division of Hainan Island. *ACTA Geogr. SINACA*. **40** (2), 169–178 (1985).

[19] Bo-Sun, W. A. N. G. et al. Diversity of tropical forest landscape-type in Hainan Island, China. *Acta Ecol. Sin.* **27** (5), 1690–1695 (2007).

[20] Long, J. Y. et al. Spatial autocorrelation analysis of Chinese provincial carbon dioxide emissions. *Ecol. Econ.*10.1109/CSO.2009.147 (2011).

[21] WEI, P. et al. Spatial and Temporal characteristics of vegetation resilience to drought in China. *Sci. China Earth Sci.* **68**(07), 2310–2327 (2025).

[22] Zhang, C., Li, S. & Wan, J. H. The warmest year 2015 in the instrumental record and its comparison with year 1998. *Atmospheric Ocean. Sci. Lett.* **9** (6), 487–494 (2016).

[23] Niinistö & Kellomäki Silvola. Seasonality in a boreal forest ecosystem affects the use of soil temperature and moisture as predictors of soil CO_2_ efflux. *Biogeosciences Discuss.* **8** (8), 2811–2849 (2011).

[24] Huimin, Y. et al. Multiple cropping intensity in China derived from Agro-meteorological observations and MODIS data. *Chin. Geogra. Sci.* **24** (02), 205–219 (2014).

[25] Doughty, C. E. & Goulden, M. L. Are tropical forests near a high temperature threshold?. *J. Geophys. Res. : Biogeosc 113* **113**, G00B07 (2008).

[26] Cox, P. M. et al. Sensitivity of tropical carbon to climate change constrained by carbon dioxide variability. *Nature* **494**, 341–344 (2013). 23389447 10.1038/nature11882

[27] Li, G. et al. Analysis and prediction of global vegetation dynamics:past variations and future perspectives. *J. Forestry Res.* **34** (02), 317–332 (2023).

[28] Tadesse, K. B. & Dinka, M. O. .Application of SARIMA model to forecasting monthly flows in Waterval River, South Africa. *J. Water Land. Dev.*10.1515/jwld-2017-0088 (2017).

[29] Aboelnour, M. A. et al. Leveraging ERA5-Land reanalysis precipitation data for urban flood vulnerability and water security assessments: A global perspective. *Earth Syst. Environ.* **9** (3), 2335–2353 (2025).

[30] GoovaertsP Kriging interpolation. *Geographic Inform. Sci. Technol. Body Knowl.*10.22224/gistbok/2019.4.4 (2019).

[31] YU Yan-sheng, C. H. E. N. & Xing-wei Analysis of future trend characteristics of hydrological time series based on R/S and Mann-Kendall methods. *J. Water Resour. Water Enigineering*. **19** (3), 4144 (2008).

